# Tyrosine Kinases and Phosphatases: Enablers of the *Porphyromonas gingivalis* Lifestyle

**DOI:** 10.3389/froh.2022.835586

**Published:** 2022-02-09

**Authors:** Richard J. Lamont, Daniel P. Miller

**Affiliations:** ^1^Department of Oral Immunology and Infectious Diseases, University of Louisville School of Dentistry, Louisville, KY, United States; ^2^Department of Microbiology and Immunology, Virginia Commonwealth University Richmond, Richmond, VA, United States

**Keywords:** tyrosine kinase, tyrosine phosphatase, *Porphyromonas gingivalis*, polymicrobial, epithelial cells

## Abstract

Tyrosine phosphorylation modifies the functionality of bacterial proteins and forms the basis of a versatile and tunable signal transduction system. The integrated action of tyrosine kinases and phosphatases controls bacterial processes important for metabolism and virulence. *Porphyromonas gingivalis*, a keystone pathogen in periodontal disease, possesses an extensive phosphotyrosine signaling network. The phosphorylation reaction is catalyzed by a bacterial tyrosine (BY) kinase, Ptk1, and a Ubiquitous bacterial Kinase UbK1. Dephosphorylation is mediated by a low-molecular-weight phosphatase, Ltp1 and a polymerase and histidinol phosphatase, Php1. Phosphotyrosine signaling controls exopolysaccharide production, gingipain activity, oxidative stress responses and synergistic community development with *Streptococcus gordonii*. Additionally, Ltp1 is secreted extracellularly and can be delivered inside gingival epithelial cells where it can override host cell signaling and readjust cellular physiology. The landscape of coordinated tyrosine kinase and phosphatase activity thus underlies the adaptive responses of *P. gingivalis* to both the polymicrobial environment of bacterial communities and the intracellular environment of gingival epithelial cells.

## Introduction: General Features of Tyrosine Phosphorylation in Bacteria

Signal transduction mediated by coordinated and reversible protein phosphorylation events regulates a wide variety of biological processes in all living cells. In bacteria, phospho-dependent signaling can occur through two component systems (TCS) which rely on His/Asp phosphorylation, as well as through phosphorylation of Ser/Thr or of Tyr residues [1, 2]. Moreover, relaxed substrate recognition by Ser/Thr/Tyr kinases and phosphatases ensures that these systems are not insulated from each other and can converge in the same circuit, increasing the plasticity of signaling [3, 4]. Tyrosine phosphorylation is catalyzed by distinct types of residue-specific kinases and is reversed by cognate phosphatases. In prokaryotes, the Bacterial Tyrosine (BY) kinases are the most extensively studied tyrosine-phosphorylating enzyme [2, 4]. The BY kinases do not have eukaryotic counterparts and possess a catalytic domain characterized by Walker ATP binding motifs and a C-terminal tyrosine cluster (YC). A hydrophobic domain directs the enzyme to the cell membrane where it can act as a sensor for environmental stimuli. However, BY kinases can also be located in the cytoplasm [5].

Substrate phosphorylation is initiated upon autophosphorylation of the BY kinase which dissociates octamers of inactive enzyme, and releases the YC of one monomer from the catalytic site of an adjacent monomer which is then available for substrate recognition [6]. More recently, the Ubiquitous bacterial Kinase (UbK) family was characterized [7]. UbK enzymes have dual specificity for both serine/threonine and tyrosine, and *ubk* genes are present in most bacterial genomes, but not in Archea or eukaryotes [7]. There are three major families of tyrosine phosphatases which dephosphorylate and thereby inactivate BY kinases: low-molecular-weight phosphatases (LMW-PTPs), small acidic enzymes also found in eukaryotes; the eukaryotic-like phosphatases (PTPs), dual-specific phosphatases that also display activity against phosphoserine and phosphothreonine; and the DNA polymerase and histidinol phosphate phosphoesterase (PHP) family [2].

Regulation of BY-kinases by phosphatases occurs through dephosphorylation of the YC. Nevertheless, BY kinase activity is not dependent simply on the phosphorylation state of one key regulatory residue, as is the case for a majority of eukaryotic kinases. Rather, activity varies between a state where the YC motif is highly phosphorylated, to a state where YC phosphorylation is significantly reduced [8], and the presence of both of these states is essential for homeostasis. BY kinases and phosphatases thus do not form classical on/off switches, but rather comprise a continuum of activity with an associated spectrum of biological function. Tyrosine phosphatases are also capable of direct substrate dephosphorylation, which together with regulation of phosphatase activity by tyrosine phosphorylation, adds additional layers of regulation [9–11].

The phosphotyrosine proteome in bacteria is quite extensive, with over 500 unique phosphotyrosine sites identified on almost 350 proteins in *E. coli* [12]. Tyrosine phosphorylation coordinates a number of important processes including heat shock responses, DNA replication, transcriptional regulation, and metabolic activity. Moreover, tyrosine kinases and phosphatases control the biosynthesis and export of polysaccharides responsible for the formation of biofilms or of capsules essential for virulence. As bacterial kinases and phosphatases are active on eukaryotic substrates, many pathogenic bacteria weaponize these enzymes to manipulate signaling in host cells and promote survival in the hosts. The action of tyrosine kinases and phosphatases, therefore, is a key regulatory node that can underlie bacterial pathogenesis in a context- and species- dependent manner.

## The *Porphyromonas Gingivalis* Lifestyle

*Porphyromonas gingivalis*, a keystone pathogen in periodontal disease, is an obligate asaccharolytic anaerobe. In the ecosystem of the subgingival compartment, *P. gingivalis* is a constituent of a complex polymicrobial community and it is this community that is the etiological unit in periodontal disease [13, 14]. Much of the physiology of *P. gingivalis* is geared toward acquisition of peptides through the action of gingipain proteases on host proteins, resistance to oxidative stress, and synergistic interactions, including metabolic communication, with community partners. In addition to interbacterial interactions, *P. gingivalis* engages gingival epithelial cells in an intricate molecular dialogue which can disrupt tissue and immune homeostasis, to create a microenvironment conducive to survival and persistence. As will be discussed, tyrosine (de)phosphorylation based signaling plays a key role in these processes. First, however, an exposition of the tyrosine kinases and phosphatases produced by *P. gingivalis*.

## The Tyrosine Kinase and Phosphatase Catalog in *P. Gingivalis*

Study of kinases and phosphatases in *P. gingivalis* has been performed mainly in strain 33277, although homologs are present in other sequenced strains.

(i) Tyrosinse kinases. (A) The Ptk1 BY kinase of *P. gingivalis* contains the canonical Walker A, A' and B domains, and the YC motif [15, 16]. In addition, an arginine- and lysine- rich RK cluster contributes to catalytic activity, which is characteristic of single polypeptide chain enzymes. In the YC domain, 6 tyrosine residues in a 16 aa region are autophosphorylated. (B) Ubk. UbK1 contains conserved SPT/S, Hanks-type HxDxYR, EW, and Walker A motifs [17]. The Walker A domain and the Hanks-type domain are necessary for both autophosphorylation and transphosphorylation. UbK1 autophosphorylates on the proximal serine in the SPT/S domain as well as the tyrosine residue within the HxDxYR domain and the tyrosine residue immediately proximal, indicating both serine/threonine and tyrosine specificity.

(ii) Tyrosine phosphatases. (A) Ltp1 is a LMW-PTP which contains the conserved catalytic motif C(X)_5_R. The nucleophile cysteine within this phosphotransfer loop is required for phosphatase activity, while the arginine is necessary for substrate binding [18, 19]. (B) Php1, a PHP family enzyme, with divalent metal ion-dependent activity [10]. Conserved histidine residues in the PHP domain are important for the coordination of metal ions, and a conserved arginine residue is necessary for catalysis. The *php1* gene is located immediately downstream of the *ptk1* gene, whereas *ltp1* is remotely located, an uncommon arrangement in Gram-negative bacteria.

## Tyrosine Kinase and Phosphatase Functions in *P. Gingivalis*

**Figure 1:**
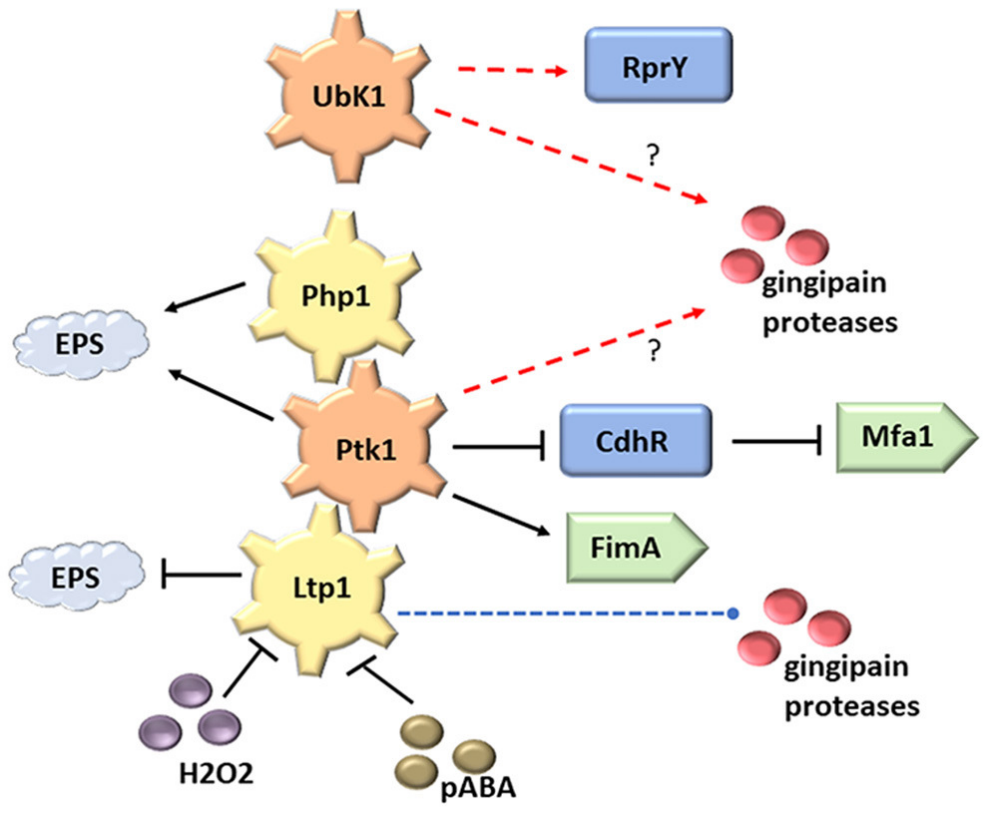
Tyrosine kinases (Ptk1 and UbK1) and phosphatases (Ltp1 and Php1) in *P. gingivalis*. Both Ltp1 and Php1 can dephosphorylate and inactivate Ptk1. RprY and CdhR are transcriptional regulators controlled by UbK1 and Php1 respectively. The Php1/Ltp1-Ptk1 axis can control production of the FimA and Mfa1 fimbriae and extracellular polysaccharide (EPS). Processing and secretion of gingipain proteases requires specific patterns of tyrosine phosphorylation. Secreted Ltp1 can be inactivated by peroxide and pABA. Red dashed line indicates direct phosphorylation and blue dashed line indicates direct dephosphorylation.

(i) Extracellular polysaccharide (EPS) production. One of the first functions described for tyrosine (de)phosphorylation in bacteria is extracellular polysaccharide production. In *E. coli*, the Wzc BY-kinase together with its cognate tyrosine phosphatase, Wzb, are responsible for group 1 capsule and colonic acid polysaccharide biosynthesis [1, 20]. The current model holds that export of EPS is driven by a ternary complex minimally comprising octameric Wzc, along with octameric open-form of the integral outer membrane translocase Wza and the polymerase Wzy. Wzc autophosphorylation causes octamer dissociation leading to closure of the Wza translocon. Reactivation then depends on the dephosphorylation of Wzc by Wzb [21]. Detailed mechanistic studies have not been performed in *P. gingivalis*; however, deletion of the *ptk1* gene results in loss of EPS secretion in strain 33277, and failure to produce a capsule in strain W83 [15]. Ptk1 can phosphorylate the capsule related proteins PGN_0224, a UDP-acetyl-mannosamine dehydrogenase, and PGN_0613, a UDP-glucose dehydrogenase [15], as also occurs in *E. coli* [22] and *Staphylococcus aureus* [23], indicating that Ptk1 may play a similar role in EPS production as the BY kinases in these organisms. Also consistent with the canonical model, a *php1* mutant of *P. gingivalis* produces significantly less EPS [10]. Interestingly, the Ltp1 tyrosine phosphatase downregulates exopolysaccharide production in *P. gingivalis*, through impacting transcriptional regulation of multiple genes involved in biosynthesis and transport [24]. Hence, EPS levels in *P. gingivalis* will depend on a balance of the activities of Ptk1, Php1 and Ltp1, a theme that pervades many of the tyrosine kinases/phosphatases signaling events.

(ii) Gingipain activity. The arginine (RgpA, RgB) and lysine (Kgp) specific gingipain proteases are critical for growth and virulence of *P. gingivalis*. These enzymes degrade host proteins, including immune effectors, to provide peptides for metabolism [25, 26]. The gingipains are also involved in maturation of several surface proteins. Although the involvement of Ptk1 and/or Ubk1 has yet to be determined, site-directed mutagenesis showed that phosphotyrosines in the hemagglutinin/adhesin domains of RgpA and Kgp, and in the catalytic domain of RgpB, influenced secretion, processing, and enzymatic activity [27]. The effects may stem from aberrant processing of latent progingipains. In RgpB and Kgp phosphorylation of tyrosines can also regulate folding and stability. Additionally, the phosphorylation status of one gingipain influenced the others, suggesting interdependence of phosphorylation-based maturation pathways. Ltp1 can also independently impact gingipain secretion and activity [24], consistent with the notion of specific roles for individual tyrosine residues.

(iii) Oxidative stress. UbK enzymes can control stress responses in other organisms [28, 29]. In *P. gingivalis*, Ubk1 phosphorylates and modulates the activity of the orphan response regulator (RR) RprY [17]. The RprY regulon in *P. gingivalis* includes genes involved in oxidative stress responses, suggestive of a role for UbK in the process. However, this contribution of Ubk1 to *P. gingivalis* physiology has yet to be confirmed, a problematical task given that *ubk1* is an essential gene in the organism [17].

(iv) Heterotypic community development. Tyrosine phosphorylation dependent signaling plays a major role in the development of synergistically pathogenic communities of *P. gingivalis* with *Streptococcus gordonii*. Co-adhesion between the organism is mediated by the FimA and Mfa1 fimbriae of *P. gingivalis* engaging the SspA/B and GAPDH surface proteins, respectively, of *S. gordonii* [30–36]. Ptk1 controls the production of both fimbrial types, in the case of Mfa1 through inactivation of CdhR, a negative transcriptional regulator of *mfa1* gene expression [24, 37] (Figure 1). Consequently, mutants of *ptk1* are deficient in *P. gingivalis*-*S. gordonii* community development [15]. Furthering the concept that both kinase and phosphatase action is required for signaling competence, loss of Php1 also impedes dual species community formation [10]. Interestingly, however, loss of Ltp1 has the opposite effect, and *ltp1* mutants show enhanced accumulation on substrata of *S. gordonii* [24]. This may be the consequence of Ltp1 have different kinetics or target residues for Ptk1 dephosphorylation, or of Ltp1 directly dephosphorylating other community functional substrates. These distinct roles of Ltp1 and Php1 contribute to the overall regulation of community development. Ltp1, but not Php1, is inhibited by extracellular metabolites of *S. gordonii* such as hydrogen peroxide (which oxidizes the catalytic cysteine and renders it unable to act as a nucleophile) and para-amino benzoic acid (pABA) [10] (Figure 1). Sensing of *S. gordonii* metabolites through inactivation of Ltp1 thus leads to increased fimbrial adhesin production and primes *P. gingivalis* for community development [38]. In this manner *P. gingivalis* may be able to sample the environmental milieu and use phosphotyrosine signaling to promote adherence and co-colonization with metabolically compatible synergistic organisms.

**Figure 2:**
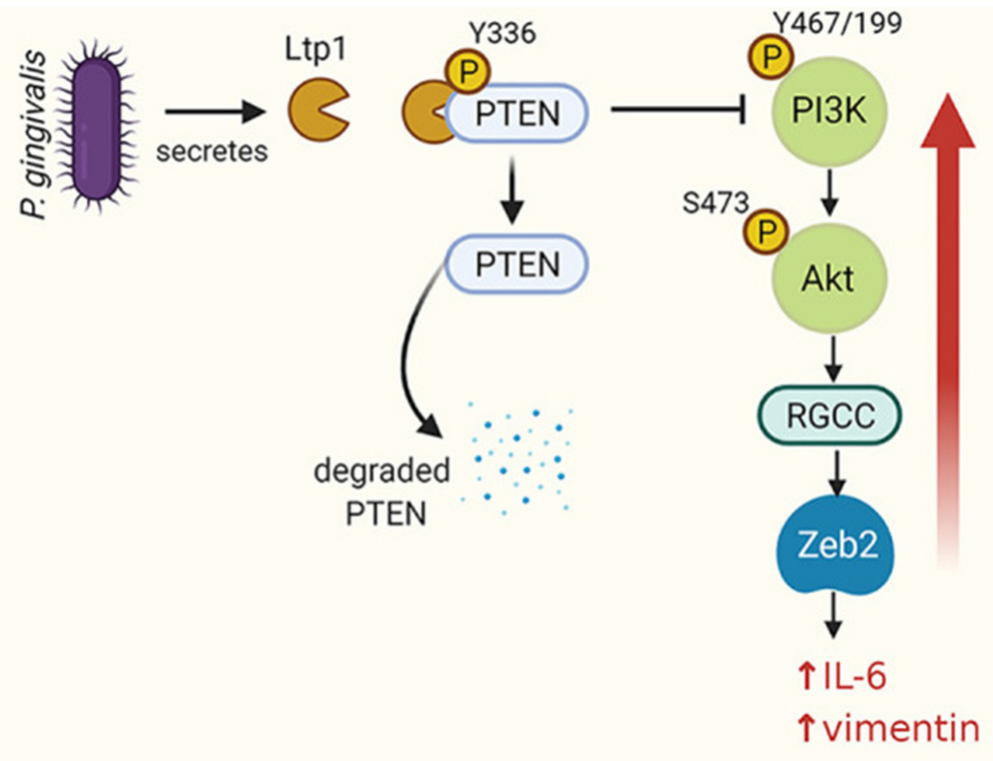
Schematic of the impact of Ltp1 secreted within epithelial cells by *P*. *gingivalis*. Ltp1 dephosphorylates PTEN leading to proteasomal degradation. A reduction of PTEN levels relieves suppression of PI3K/Akt which upregulates RGCC and Zeb2. An increase in Zeb2 activity results in upregulation of IL-6 and vimentin. From Liu et al. [44].

(v) Epithelial cell manipulation. Eukaryotic LMW-PTPs have emerged as signaling hubs in a number of physiological processes and are drivers of carcinogenesis and cancer aggressiveness [39]. Bacterial LMW-PTPs are also operational within host cells. Enteric pathogens such as *Salmonella* and *Yersinia* can deliver tyrosine phosphatases into epithelial cells through the Type III secretion machinery [18, 40, 41]. Intracellularly, these bacterial enzymes act on host cell phosphoproteins to uncouple signal transduction pathways and contribute to persistence and virulence. Bacterial LMW-LTPs can also be secreted by yet to be defined mechanisms [42, 43], and in the case of PtpA of *Mycobacterium tuberculosis*, enter into host cell nuclei [43]. *P. gingivalis* can also introduce Ltp1 directly into gingival epithelial cells where it can translocate to the nucleus [44]. As Ltp1 lacks the consensus sequence for recognition by the Type IX secretion system, the secretion process remains to be established. An intracellular location will protect the enzyme from inactivation by metabolites such as pABA and peroxide produced by other organisms in the extracellular environment. Intracellular Ltp1 dephosphorylates PTEN on Y336 which results in elevated proteasomal degradation [44]. Reduced PTEN levels cause an increase in signaling through the PI3K-Akt pathway which converges on transcriptional upregulation of Regulator of Growth and Cell Cycle (RGCC) (Figure 2). The epithelial-mesenchymal transition (EMT) transcriptional regulator Zeb2 is controlled by RGCC and thus Ltp1 can increase expression of mesenchymal markers and enhance epithelial cell migration and proliferation. Ltp1 also increases production of proinflammatory cytokines such as IL-6. Ltp1 thus contributes to a protumorigenic phenotype in epithelial cells and a proinflammatory microenvironment.

## Conclusions

Signaling coherence is enhanced when circuitry is tunable and can be insulated from, or interconnected with, other transduction mechanisms. Bacterial protein tyrosine phosphorylation/dephosphorylation illustrates this principle. Extending beyond simple on/off switches, the kinases and phosphatases are structurally geared to cycle between high and low activity states by reciprocating phosphorylation and dephosphorylation reactions. In *P. gingivalis*, the presence of two phosphatases may allow additional calibration of the Ptk1 BY kinase. *P. gingivalis* exploits the efficiency and precision of phosphotyrosine dependent signaling to impose layers of control over functions important for in vivo colonization and persistence. Sensitivity of the Ltp1 LMW-PTP to extracellular metabolites creates a sensing system for detection of potential community partners and upregulation of relevant adhesins. Delivery of Ltp1 within gingival epithelial cells, where it can reprogram host signal transduction pathways, has consequences for host cell, tissue and inflammatory homeostasis. The emergence of phosphotyrosine signaling as a key device in the control of numerous cellular functions in *P. gingivalis* creates access points for novel therapeutics designed to maintain the organism in a state of eubiosis and prevent dysbiotic interactions with the host.

## Author Contributions

RL and DM wrote the manuscript and approved the submitted version.

## Funding

We thank the NIH for supporting the original research that is the basis for this review through DE023193, DE012505, DE011111 (RL), and DE028346 (DM). The funding sources had no input in study design; in the collection, analysis and interpretation of data; in the writing of the report; or in the decision to submit the article for publication.

## Conflict of Interest

The authors declare that the research was conducted in the absence of any commercial or financial relationships that could be construed as a potential conflict of interest.

## Publisher's Note

All claims expressed in this article are solely those of the authors and do not necessarily represent those of their affiliated organizations, or those of the publisher, the editors and the reviewers. Any product that may be evaluated in this article, or claim that may be made by its manufacturer, is not guaranteed or endorsed by the publisher.
